# Brain grey and white matter structural associations with *future* suicidal ideation and behaviors in adolescent and young adult females with mood disorders

**DOI:** 10.1002/jcv2.12118

**Published:** 2022-11-21

**Authors:** Lejla Colic, Luca M. Villa, Maria R. Dauvermann, Laura S. van Velzen, Anjali Sankar, Danielle A. Goldman, Priyanka Panchal, Jihoon A. Kim, Susan Quatrano, Linda Spencer, R. Todd Constable, John Suckling, Ian M. Goodyer, Lianne Schmaal, Anne-Laura van Harmelen, Hilary P. Blumberg

**Affiliations:** 1 Department of Psychiatry, Yale School of Medicine, New Haven, Connecticut, USA; 2 Department of Psychiatry and Psychotherapy, Jena University Hospital, Jena, Germany; 3 German Center for Mental Health, Jena, Germany; 4 Department of Psychiatry, University of Oxford, Oxford, UK; 5 Department of Psychiatry, University of Cambridge, Cambridge, UK; 6 McGovern Institute for Brain Research, Massachusetts Institute of Technology, Cambridge, Massachusetts, USA; 7 Institute for Mental Health, School of Psychology, University of Birmingham, Birmingham, UK; 8 Orygen, Parkville, Victoria, Australia; 9 Centre for Youth Mental Health, The University of Melbourne, Parkville, Victoria, Australia; 10 Department of Neurology and Neurobiology Research Unit, Copenhagen University Hospital, Kobenhavn, Denmark; 11 Interdepartmental Neuroscience Program, Yale School of Medicine, New Haven, Connecticut, USA; 12 Department of Radiology and Biomedical Imaging, Yale School of Medicine, New Haven, Connecticut, USA; 13 Institute of Education and Child Studies, Leiden University, Leiden, The Netherlands; 14 Child Study Center, Yale School of Medicine, New Haven, Connecticut, USA

**Keywords:** adolescence, gender, magnetic resonance imaging, mood disorder, suicidal behaviour

## Abstract

**Background:**

To reduce suicide in females with mood disorders, it is critical to understand brain substrates underlying their vulnerability to future suicidal ideation and behaviors (SIBs) in adolescence and young adulthood. In an international collaboration, grey and white matter structure was investigated in adolescent and young adult females with future suicidal behaviors (*f*SB) and ideation (*f*SI), and without SIBs (*f*nonSIB).

**Methods:**

Structural (*n* = 91) and diffusion-weighted (*n* = 88) magnetic resonance imaging scans at baseline and SIB measures at follow-up on average two years later (standard deviation, SD = 1 year) were assessed in 92 females [age(SD) = 16.1(2.6) years] with bipolar disorder (BD, 28.3%) or major depressive disorder (MDD, 71.7%). One-way analyses of covariance comparing baseline regional grey matter cortical surface area, thickness, subcortical grey volumes, or white matter tensor-based fractional anisotropy across *f*SB (*n* = 40, 43.5%), *f*SI (*n* = 33, 35.9%) and *f*nonSIB (*n* = 19, 20.6%) groups were followed by pairwise comparisons in significant regions (*p* < 0.05).

**Results:**

Compared to *f*nonSIBs, *f*SIs and *f*SBs showed significant decreases in cortical thickness of right inferior frontal gyrus pars orbitalis and middle temporal gyrus, *f*SIs of left inferior frontal gyrus, pars orbitalis. *F*SIs and *f*SBs showed lower fractional anisotropy in left uncinate fasciculus and corona radiata, and *f*SBs in right uncinate and superior fronto-occipital fasciculi.

**Conclusions:**

The study provides preliminary evidence of grey and white matter alterations in brain regions subserving emotional and behavioral regulation and perceptual processing in adolescent and young adult females with mood disorders with, versus without, future SIBs. Findings suggest potential targets to prevent SIBs in female adolescents and young adults.

## INTRODUCTION

Suicide is the second leading cause of death for adolescents and young adults worldwide (Glenn et al., 2020). Adolescence is the epoch when suicidal ideation (SI) and behaviors (SBs) typically emerge (Nock et al., 2013). Although adolescent onset of SI and SBs confers risk for future SI and SBs (Copeland et al., 2017), there is substantial individual variation in the future risk (Goldston et al., 2016). Identification of brain structural vulnerabilities associated with future SI and SBs could provide important targets for prevention strategies in persons at highest risk. Individuals suffering from mood disorders, including bipolar disorder (BD) and major depressive disorder (MDD), comprise the majority who die by suicide (Chesney et al., 2014). Thus, findings associated with future suicidal ideation and behaviors (SIBs) across these diagnoses could lead to targets for prevention for a large group of individuals at risk.

Brain development during adolescence and young adulthood is characterized by maturation of grey matter in the frontal cortex and its major projection sites, and the white matter of the connections between them, which show alterations in persons suffering from mood disorders (Kelberman et al., 2020). As these regions overlap with some of those implicated in SIBs, there may be a link between part of the grey and white matter structures characterizing adolescents and young adults with mood disorders and SIB development. However, as recently identified in a review by Schmaal et al. (2020) there are notable research gaps; much of the neuroimaging research on SIBs has focused on adult participants, thus, it is not known whether structural vulnerabilities are present also in adolescents. Sample sizes in the few prior neuroimaging studies of SIBs in adolescents and young adults were small, limiting ability to compare individuals with and without SIBs. In addition, in neuroimaging studies of individuals at any age, prospective measures of SIBs were rare so that little is known about associations between brain structure and future SIBs.

Recent findings from the small number of magnetic resonance imaging (MRI) studies in combined samples of female and male adolescents and young adults with mood disorders support associations between lifetime suicide attempts and altered brain structure in ventral and dorsal frontal as well as temporal cortex, striatal and limbic areas (Fan et al., 2019; Gifuni et al., 2021; Ho et al., 2021; Hong et al., 2021; Huber et al., 2019; Johnston et al., 2017; Pan et al., 2015), and white matter tracts that connect them (Auerbach et al., 2020; van Heeringen et al., 2014). However, these studies primarily used retrospective reports of SBs and had limited information on prior SI. The study of SI is important in order to generate strategies to prevent suffering, identify at early stages who is at most risk to have future SI and SBs (Klonsky et al., 2016) and interrupt the potential transition to suicide attempt. To the best of our knowledge, there is only one prior report on the association between altered brain structure and prospective measures of future suicide attempts in younger persons with mood disorders (Lippard et al., 2019). This study included 17 adolescents and young adults with BD and MDD who attempted suicide during a period of one to five years and showed lower grey matter volume in ventral and rostral frontal cortices, and lower fractional anisotropy in dorsal frontal tracts, internal capsule and cingulum. These brain regions and tracts are associated with dysregulated behaviors known to increase suicide risk, including emotion dysregulation (Hatkevich et al., 2019) and impaired ability to inhibit other non-adaptive behaviors (Rudd, 2000), suggesting that differences in brain structure may underlie future risk for suicide attempts. This study, as well as most prior structural imaging studies of SIBs, assessed potential differences in volume measures. As cortical surface area and thickness differ in genetic and developmental mechanisms (Grasby et al., 2020), the study of their specific associations to SI and SBs could be an important step in elucidating underlying neuropathophysiological processes.

There are indications that SIBs have distinct mechanisms between the adolescent females and males. For example, a national survey in US indicated that SI and SBs are reported by 15% and 6% of female youth, respectively, compared to 9% and 2% of male youth (Nock et al., 2013), and a study in Korea indicated 19% of girls report SI compared to 11% of boys (Park, 2013). Lifetime SI and SBs during adolescence may be an especially important factor for future attempts in female adolescents and young adults (Lewinsohn et al., 2001). Structural brain measures during adolescence, including cortical surface area, cortical thickness and fractional anisotropy, also differ by gender (Foulkes & Blakemore, 2018; Simmonds et al., 2014) and mood disorders have prevalence rates and clinical phenotypes that differ by gender (Diflorio & Jones, 2010; Piccinelli & Wilkinson, 2000).

To address limitations of previous neuroimaging studies of SIBs, in this study we investigated associations of grey matter cortical surface area and thickness, subcortical grey volume, and white matter fractional anisotropy, to future SI and SBs across adolescent and young adult females with BD or MDD. In this first study of its kind, data was combined from independent samples at two international sites. Given the differences between female and male adolescents and young adults, our study focused on investigating prospective SI and SBs only in female participants to improve the sample homogeneity. Moreover, the number of male participants was low and did not allow for direct comparison to female participants.

We hypothesized reductions in structural measures in frontal and temporal cortices, limbic and striatal projection sites, and white matter tracts that connect them (Auerbach et al., 2020; Schmaal et al., 2020), in females with *future* (*f*)SI and *f*SBs compared to without, and that reductions in frontal regions associated with emotion regulation and adaptive behavior may be found especially in the *f*SBs.

## METHODS

### Participants

Two sites, Yale University (USA) and the University of Cambridge (UK; MR-IMPACT sample, Hagan et al., 2013), contributed to data in total of 92 female participants, who met Diagnostic and Statistical Manual-IV (DSM-IV) (First et al., 1994) criteria for BD or MDD at Yale site and MDD at Cambridge site (at scanning age range 12–25.5 years, mean (standard deviation, SD) = 16.1 (2.6) years; time between visits range 1–5.5 years, mean (SD) = 2 (1) years; 28.3% BD). All participants’ past lifetime SI and SBs prior to scanning, and interim SI and SBs in the interval between assessments, were evaluated by trained staff using the Columbia-Suicide Severity Rating Scale (C-SSRS) (Posner et al., 2008). C-SSRS has good validity, high sensitivity and specificity for SBs, and is sensitive to change over time (Kelly Posner et al., 2011). Demographic and inclusion criteria for each site are described in Supporting Information S1. All participants ≥18 years of age provided written informed consent, while all participants <18 years provided written informed assent and their parent/guardian provided written informed permission/consent. The studies were approved by the Yale School of Medicine Human Investigation Committee/Institutional Review Board, and by the National Research Ethics Service Committee East of England- Cambridge Central, respectively for each site.

Using information from the C-SSRS, participants were assigned to a *f*SB group, *f*SI group or a group without *future* SI or SBs (*f*non-SIBs). The *f*SB group included participants who were evaluated to have had an actual (*n* = 31), aborted (*n* = 3) or interrupted (*n* = 2) attempt or preparatory acts (*n* = 4) in between visits (i.e., they endorsed *yes* for any item in the section of “Suicidal behavior” in C-SSRS). The other forms of SBs were included in addition to actual attempts as clarifying intent sufficient to clearly establish actual attempt criteria in youth can be challenging and engaging in these other types of SBs are associated with high risk for future actual attempts (Conway et al., 2017). The *f*SI group included participants who were evaluated to have had passive or active SI in between visits, but no SB (i.e., they endorse *yes* for any of five items in the section of “Suicidal ideation” in C-SSRS). The *f*nonSIBs group included participants who were evaluated to have had no interim SI or SB. Each group included participants who have reported lifetime SI and/or SB, that is, endorsed yes for SI or SB on C-SSRS at the baseline evaluation and the groups were compared for lifetime SI and SBs (Table 1).

Ninety-one female participants were included in the structural MRI (sMRI) analysis (Table S1); one participant was excluded from the analysis due to high motion. Eighty-eight female participants were included in the diffusion weighted tensor imaging (DTI) analysis (Table S1); four participants were excluded from the analysis due to motion artifacts.

### Magnetic resonance imaging acquisition

SMRI scans were acquired using 3 T scanners. High-resolution T1-weighted sequences, using a three-dimensional magnetically prepared rapid acquisition gradient echo sequence (3D-MPRAGE), and whole brain DTI scans were acquired in the same scanning session for each participant (details in Supporting information). All images were without neurological structural abnormalities, confirmed by consulting radiologists specializing in neuroanatomy.

### Magnetic resonance imaging processing

SMRI data were preprocessed and quality control was performed at each site according to the Enhancing NeuroImaging Genetics through Meta-Analysis (ENIGMA) consortium processing protocols (http://enigma.ini.usc.edu/protocols/imaging-protocols/) using fully automated software FreeSurfer V6 (http://surfer.nmr.mgh.harvard.edu/) (Fischl, 2012). Data were segmented for cortical surface area and thickness with the Desikan-Killany Atlas (Desikan et al., 2006) and for subcortical grey volumes with the Aseg Atlas (Desikan et al., 2006). Segmentation was visually inspected at each site using standardized protocols. Regions of interest (ROIs) and tracts for the analyses were based on a recent comprehensive review of imaging studies in SIBs (Schmaal et al., 2020). ROIs for cortical surface area and thickness were: left and right frontal pole, medial orbitofrontal, lateral orbitofrontal, inferior frontal gyrus pars orbitalis, inferior frontal gyrus pars triangularis, inferior frontal gyrus pars opercularis, superior frontal, rostral middle frontal, caudal middle frontal, rostral anterior cingulate, caudal anterior cingulate frontal pole, posterior cingulate, superior temporal gyrus, middle temporal gyrus, temporal pole and insula; for subcortical grey volume analysis: left and right amygdala, hippocampus, caudate, putamen, thalamus.

DTI data were processed according to the ENIGMA DTI protocols (http://enigma.ini.usc.edu/ongoing/dti-working-group/) and quality control was performed at each site. During pre-processing, images were eddy current corrected and skull stripped using FSL (Jenkinson et al., 2012), and bvec files were rotated accordingly. To correct for distortions, each image’s b0 volume was linearly registered to its corresponding skull stripped, T1-weighted, 3D MPRAGE image, using FSL’s FLIRT with 9° of freedom. B0 images were then non-linearly registered to their corresponding T-weighted MPRAGE images, using Advanced Normalization Tools (ANTs) (Avants et al., 2009). The deformation fields from these non-linear registrations were then applied to the DTI images. DTI images were then processed using FSL’s DTIFIT (Behrens et al., 2003, 2007), to generate FA and V1 maps. The ENIGMA- tract-based spatial statistics (TBSS) protocol, which is based on the FSL-TBSS pipeline (Smith et al., 2006), was then followed to produce skeletonized fractional anisotropy (FA) maps. Mean FA values were extracted from tracts of interest: left and right uncinate fasciculus, corona radiata, internal capsule, external capsule, inferior fronto-occipital fasciculus, superior fronto-occipital fasciculus, and corpus callosum and cingulum.

### Data analysis

Baseline demographic and lifetime SI and SB were compared between groups using Kruskal-Wallis or chi-square (*χ*^2^) tests. Outliers in imaging measures were detected using the Rosner test and excluded from the analysis. There were outliers detected and removed in sMRI measures of cortical surface area (one participant in left and right inferior frontal gyrus pars triangularis, left caudal anterior cingulate, left temporal pole; two participants in left inferior frontal gyrus pars opercularis), cortical thickness (one participant in right frontal pole), and subcortical grey volume (one participant in left and right caudate, left putamen, left and right thalamus; two participants in left amygdala). There were outliers detected and removed in DTI fractional anisotropy measures (one participant in left inferior fronto-occipital fasciculus; three participants in right corona radiata, left and right internal capsule, left external capsule, right superior fronto-occipital fasciculus, corpus callosum). Regions of interest for each modality were examined with three-group, one-way analysis of co-variance (ANCOVA). Age, site and intracranial volume (ICV; for subcortical grey volume and cortical surface area analysis) were used as covariates. Significance was set at *p* < 0.05 uncorrected, and also assessed at *p* < 0.05 corrected with false discovery rate (p_FDR_) (Benjamini & Hochberg, 1995), for the number of regions within each imaging modality. *Post-hoc* multiple pairwise-comparisons between the means of groups (*f*SB, *f*SI, *f*nonSIB) were performed using Tukey Honest Significant Differences and results were considered significant at *q* < 0.05, corrected for the three comparisons. Effect sizes were calculated with partial omega-square (*ω*_*p*_^2^; 0.01 = small, 0.06 = medium, 0.14 = large effect size) for ANCOVAs and with Hedges’ *g* (0.2 = small, 0.5 = medium, 0.8 = large effect size) for *post-hoc* pairwise-comparisons (adjusted for covariates). Additional models with age as a quadratic term and past lifetime SB as covariates were run. Analyses and graphics were run in R version 3.6.3.

## RESULTS

### Demographic and SIBs characteristics

There were no differences in age, time between visits, site, or diagnosis between the *f*SB (*n* = 40, 43.5%), *f*SI (*n* = 33, 35.9%) and *f*nonSIB (*n* = 19, 20.6%) groups. Each group had participants with past lifetime SB or SI (*f*SBs with baseline SB (yes/no) = 28/12 and SI = 37/2; *f*SIs with basline SB = 20/12 and SI = 26/3; *f*nonSIBs with baseline SB = 5/14 and SI = 8/11; Table 1). However, presence of past lifetime SBs were significantly higher (*p* = .005) in *f*SBs (70%) and *f*SIs (62.5%) compared to *f*nonSIBs (26.3%), as was past lifetime SI (*p* < .001; *f*SBs, 94.9%; *f*SIs, 89.6%; *f*nonSIBs, 42.1%), but *f*SBs and *f*SIs did not differ from each other (Table 1). All *f*SBs reported interim SI.

### SMRI measures

#### Cortical surface area

ANCOVAs were significant in right caudal middle frontal [*F*(2,85) = 3.6, *p*_uncorrected_ = .032, *ω*_*p*_^2^ = 0.054] and left middle temporal gyrus [*F*(2,85) = 3.1, p_uncorrected_ = .05, ω_p_^2^ = 0.044]. In right caudal middle frontal, *post-hoc* tests revealed significantly higher surface area in *f*SIs compared to *f*SBs (q = .04, g = 0.64), but surface area did not differ significantly between either *f*SIs or *f*SBs and *f*nonSIBs.

#### Cortical thickness

ANCOVAs were significant in left and right inferior frontal gyrus pars orbitalis [left, F(2,86) = 4.4, *p*_uncorrected_ = 0 .015, ω_p_^2^ = 0.069; right, F(2,86) = 5.3, *p* = 0 .007, ω_p_^2^ = 0.087], and right middle temporal gyrus [F(2,86) = 4.1, *p*_uncorrected_ = 0 .019, ω_p_^2^ = 0.065]. *Post-hoc* tests revealed lower cortical thickness in *f*SIs compared to *f*nonSIBs (*q* = 0 .01, *g* = 0.86) in left inferior frontal gyrus pars orbitalis, and lower thickness in right in both *f*SBs and *f*SIs compared to *f*nonSIBs (*q* = 0 .02, *g* = 0.72 and *q* = 0 .008, *g* = 0.96, respectively). In right middle temporal gyrus *f*SIs had lower thickness compared to *f*nonSIBs (*q* = 0.02, *g* = 0.79).

**FIGURE 1:**
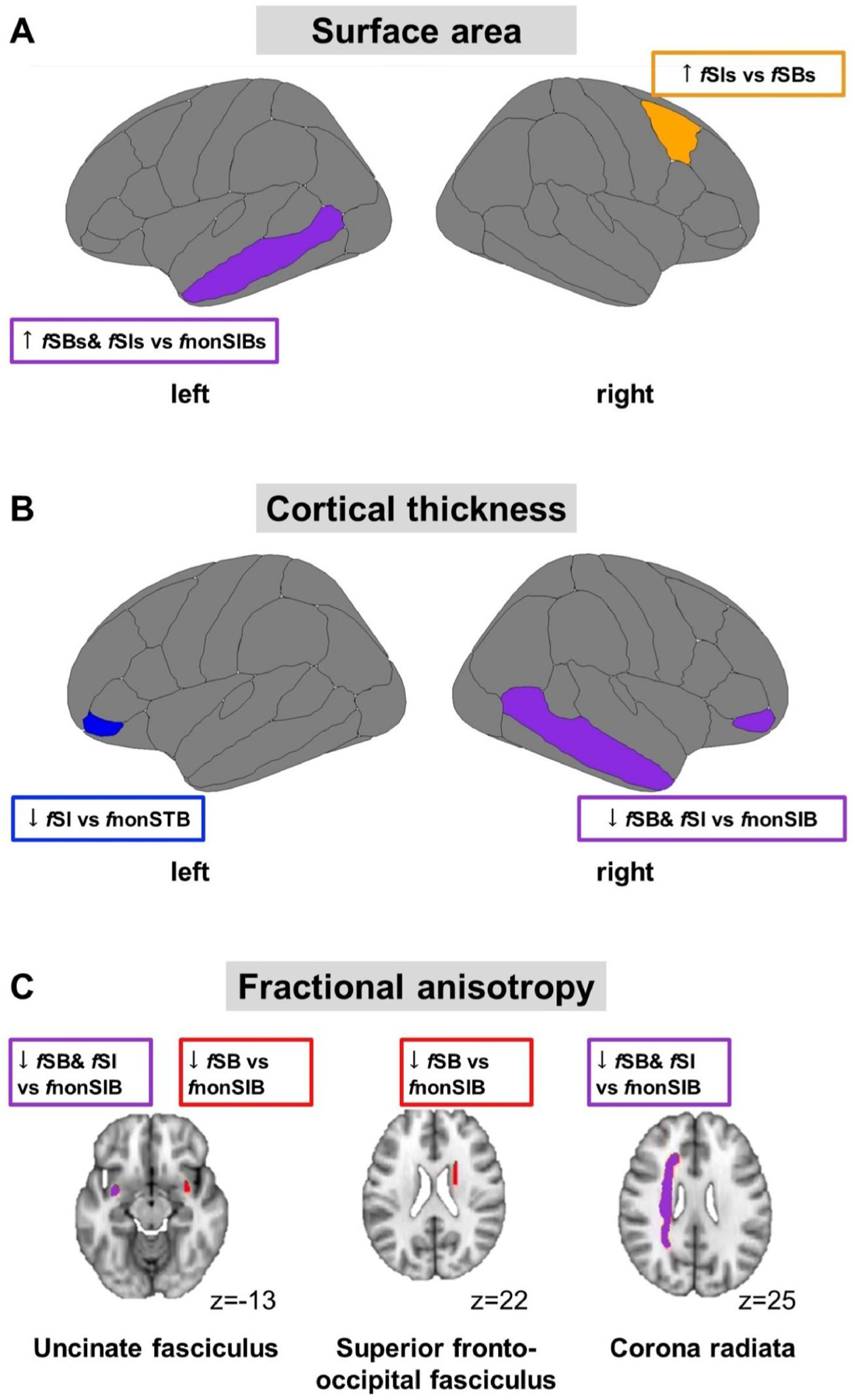
Summary figure. (A) Shows differences in cortical surface area; (B) in cortical thickness; (C) in fractional anisotropy. fnonSIBs, group with no future suicidal ideation and behaviors; fSBs, group with future suicidal behaviors; fSIs, group with future suicidal ideation.

Significant results are illustrated in Figure 1, and full results are in Table S2–S4. None of the sMRI ANCOVA results survived FDR correction.

### DTI measure

#### Fractional anisotropy

ANCOVAs were significant in bilateral uncinate fasciculus (left *F*[2,83] = 4.1, *p*_uncorrected_ = 0.019, ω_p_^2^ = 0.067; right *F*[2,83] = 3.5, *p*_uncorrected_ = 0.034, ω_p_^2^ = 0.054), left corona radiata (*F*[2,83] = 3.9, *p*_uncorrected_ = 0.023, ω_p_^2^ = 0.063) and right superior fronto-occipital fasciculus (*F*[2,80]= 4.1, *p*_uncorrected_ = 0.021, *ω*_*p*_^2^ = 0.067). In these tracts, *f*SBs showed lower FA compared to *f*nonSIBs (left uncinate fasciculus, *q* = 0.02, *g* = 0.77; right uncinate fasciculus, *q* = 0.03, *g* = 0.77; left corona radiata, *q* = 0.04, *g* = 0.82, right superior fronto-occipital fasciculus, *q* = 0.02, *g* = 0.83), while *f*SIs showed lower FA in left uncinate fasciculus (*q* = 0.04, *g* = 0.76) and left corona radiata (*q* = 0.03, *g* = 0.68) compared to *f*nonSIBs.

Significant results are illustrated in Figure 1 and the full results are in Table S5. None of the DTI ANCOVA results survived FDR correction.

The models with quadratic age term remained on the same level of significance, thus the additional term was dropped for parsimony. SMRI and DTI results had similar level of significance when covariaed for past lifetime SBs (Table S6).

## DISCUSSION

This study examined associations between brain structure and *future* SIBs across two independent samples of adolescent and young adult females suffering from mood disorders, scanned at baseline and divided into *f*SB, *f*SI and *f*nonSIB groups based on their C-SSRS assessments 1–5.5 years after the initial scanning visit. Consistent with the high prevalence of SIBs in youth suffering from mood disorders, especially females (Nock et al., 2013), the proportion with *future* SIBs was high (79.3%), underscoring the urgent need for better understanding of the neurobiology underlying SIBs during this critical developmental period. Although cortical grey matter surface area and thickness and subcortical grey matter were studied, grey matter structural vulnerabilities were primarily found in cortical thickness and were accompanied by findings in white matter fractional anisotropy. Both cortical thickness and white matter undergo maturational changes during adolescence but with unique neurobiological processes contributing to each (Tamnes et al., 2010). Significant imaging findings of this study included, compared to *f*nonSIBs, decreases in *f*SIs and *f*SBs in right inferior frontal gyrus pars orbitalis cortical thickness and left uncinate fasciculus and left corona radiata fractional anisotropy. In addition, *f*SIs showed lower cortical thickness in left inferior frontal gyrus pars orbitalis and right middle temporal gyrus cortical thickness compared to *f*nonSIBs. *F*SBs showed lower fractional anisotropy in right uncinate and superior fronto-occipital fasciculi, compared to *f*nonSIBs.

Previous studies reported lower thickness in similar ventrolateral prefrontal regions to the inferior frontal gyrus pars orbitalis in youth with BD and MDD and lifetime SIBs (Hong et al., 2021; Huber et al., 2019). These brain regions subserve adaptive behavior in the setting of changing reinforcement contingencies (Ghahremani et al., 2010), domains in which dysfunction has been theorized to contribute to SIBs. Differences in cortical thickness were also found in right middle temporal gyrus, a region that was reported to have lower thickness in adults with mood disorders who were recent suicide attempters (Kim et al., 2021). This temporal area processes perceptual information and has frontal connections and could play a role in adaptive integration of sensory information (Sekiyama et al., 2003); however, the area has also been implicated in psychotic symptoms that can increase SIBs risk (Giakoumatos et al., 2013). These initial findings of the associations with *future* SIBs are important in providing support for the role of these brain regions and the behaviors they subserve in SIB vulnerability. Cortical thickness is thought to be shaped by the environment and processes that occur after early childhood, whereas cortical surface area has higher genetic heritability and seems to be shaped during prenatal and early post-natal periods (Grasby et al., 2020). In addition to cortical thickness findings, *f*SIs had higher surface area than *f*SBs in right caudal middle frontal gyrus.

Fractional anisotropy was lower in white matter that carries projections from and to frontal and temporal regions where we observed thickness decreases. For example, the decreases in the uncinate fasciculus, a major fibre bundle providing ventral frontal to temporal connections that subserve emotion regulation (Von Der Heide et al., 2013), are consistent with previous reports by our group in adolescents and young adults with mood disorders and past suicide attempts (Fan et al., 2019; Johnston et al., 2017; Lippard et al., 2019). We show that our findings are associated with *future* SIBs and that while there are left uncinate reductions in both *f*SBs and *f*SIs, they were present in the right hemisphere only in *f*SBs. In addition to uncinate findings, both *f*SBs and *f*SIs showed reductions in fractional anisotropy in the corona radiata. Findings in this tract have been reported in adults with mood disorders and prior suicide attempts (Wei et al., 2020). The corona radiata is part of thalamic-cortical circuitry that has been associated with behavioral regulation (Jenkins et al., 2016). We also observed decreases in *f*SBs in the right superior fronto-occipital fasciculus, which provides frontal connections to posterior sensory areas and may be involved in the integration of perceptual information into behavioral action (Schmahmann & Pandya, 2007).

Neuroimaging research on past and especially future SIBs in adolescents and young adults is scarce; this study provides preliminary and hypotheses generating results. However, several limitations should be noted. Due to modest sample size, results are reported at an uncorrected threshold to balance the statistical accuracy on one hand and premature strict correction that may limit report of findings on the other. While medium to large effect sizes were observed, these results need to be replicated in future studies with larger sample sizes. While there was no difference in mean age between the groups and age was controlled for in the analysis, interaction effects between age and *future* SIBs were not investigated due to limited power. Future studies should also be designed to have consistent time interval to follow-up. The sites were not balanced for diagnosis; future studies should balance diagnostic sampling across sites to explore for potential interactions between *f*SIBs and diagnostic groups. Participants were grouped in *future* SIB groups irrespective of presence or absence of lifetime SIBs at baseline and there was a high covariation between past lifetime SIBs and *f*SIBs. Although results were similar when covaried for lifetime SBs, it is possible that prior SIBs may have influenced some of the results. We suggest that future studies in larger samples are needed to further assess potential effects of prior SIBs to disentangle cumulative effects of lifetime SIBs.

## CONCLUSIONS

Whereas previous studies focused on past lifetime SBs, this study provides preliminary findings identifying structural brain vulnerabilities associated with *future* SI and SBs in adolescent and young adult females suffering from mood disorders, a population that comprises a large proportion of persons who die by suicide in that age epoch. Further study of alterations in these brain structures, as well as the behaviors they subserve, are suggested for the generation of optimized strategies for early risk detection. In addition, research on effective interventions to reduce risk by targeting these brain structures, through pharmacological, neurostimulation or psychotherapy approaches, with potential to alter brain plasticity are urgently needed (Sankar et al., 2021).

## Supplementary Material

supinfo

## Figures and Tables

**TABLE 1:** Demographic, past suicidal ideation and behaviors, and scanning data characteristics of the three groups.

	Modality	*f*SBs	*f*SIs	*f*nonSIBs	Statistical comparison
Sample size	Total	40 (43.5%)	33 (35.9%)	19 (20.6%)	
	sMRI	40 (43.9%)	32 (35.2%)	19 (20.9%)	
	DTI	37 (42%)	33 (37.5%)	18 (20.5%)	
Age mean (SD) [range]	Total	15.6 (2.2) [12–24]	16.3 (2.7) [12–25.5]	17 (2.8) [14–25.4]	*χ*^2^(2) = 5.09, *p* = .08
	sMRI	15.6 (2.2) [12–24]	16.3 (2.8) [12–25.5]	17 (2.8) [14–25.4]	*χ*^2^(2) = 4.99, *p* = .08
	DTI	15.7 (2.3) [12–24]	16.3 (2.7) [12–25.5]	17.1(2.8) [14–25.4]	*χ*^2^(2) = 3.82, *p* = .15
Between visits time mean (SD) [range]	Total	2 (1) [1–5.5]	2.1 (1) [1–5.3]	2(0.9) [1–4.9]	*χ*^2^(2) = 2.52, *p* = .28
	sMRI	2 (1) [1–5.5]	2.1 (1) [1–5.3]	2 (0.9) [1–4.9]	*χ*^2^(2) = 2.25, *p* = .32
	DTI	2 (1.1) [1–5.5]	2.1 (1) [1–5.3]	2.1(1) [1–4.9]	*χ*^2^(2) = 2.26, *p* = .32
Yale site; *n* (%)	Total	11 (27.5%)	13 (39.4%)	7 (36.8%)	*χ*^2^(2) = 1.25, *p* = .53
	sMRI	11 (27.5%)	12 (37.5%)	7 (36.8%)	*χ*^2^(2) = 0.97, *p* = .62
	DTI	11 (29.7%)	13 (39.4%)	7 (38.9%)	*χ*^2^(2) = 0.85, *p* = .65
Bipolar disorder; *n* (%)	Total	10 (25%)	12 (36.4%)	4 (21%)	*χ*^2^(2) = 1.76, *p* = .41
	sMRI	10 (25%)	12 (37.5%)	4 (21%)	*χ*^2^(2) = 2.03, *p* = .36
	DTI	10 (27%)	12 (36.4%)	4 (22.2%)	*χ*^2^(2) = 1.31, *p* = .52
Past lifetime suicidal behaviors yes; *n* (%)	Total	28 (70%)	20 (62.5%)^a^	5 (26.3%)	*χ*^2^(2) = 10.47, *p* = .005**
	sMRI	28 (70%)	20 (64.5%)^a^	5 (26.3%)	*χ*^2^(2) = 10.77, *p* = .004**
	DTI	26 (70.3%)	20 (62.5%)^a^	4 (22.2%)	*χ*^2^(2) = 11.96, *p* = .002**
Past lifetime suicidal ideation yes; *n* (%)	Total	37 (94.9%)^a^	26 (89.6%)^b^	8 (42.1%)	*χ*^2^(2) = 25.58, *p* < .001***
	sMRI	37 (94.9%)^a^	26 (92.8%)^b^	8 (42.1%)	*χ*^2^(2) = 27.76, *p* < .001***
	DTI	34 (94.4%)^a^	26 (89.6%)^b^	7 (38.9%)	*χ*^2^(2) = 26.09, *p* < .001***

*Note*: Groups were compared with Kruskal-Wallis for continuous variables or chi-square test for nominal variables.

Abbreviations: DTI, diffusion-weighted tensor imaging; fnonSIBs, group with no future suicidal ideation and behaviors; fSBs, group with future suicidal behaviors; fSIs, group with future suicidal ideation; *n*, number of participants; SD, standard deviation; sMRI, structural magnetic resonance imaging.

a Missing information for one participant.

b Missing information for four participants.

## Data Availability

The data that support the findings of this study are available on request from senior authors.

## Key points

- To address high rates of in suicide ideation and behaviors in female adolescents and young adults with mood disorders, it is critical to identify associated brain alterations.
- In an international consortium, grey and white matter brain structure was investigated in female adolescents and young adults with mood disorders with a focus on longitudinal (future) suicide ideation and behaviors.
- Females with future suicide ideation and behaviors showed decreases in cortical thickness and fractional anisotropy in regions and tracts subserving emotional and behavioral regulation.
- These preliminary findings may aid in generating targeted interventions through pharmacological, neurostimulation or psychotherapy approaches.
