# Supplementary material for: Brain grey and white matter structural associations with future suicidal ideation and behaviors in adolescent and young adult females with mood disorders
Source: JCPP Adv. 2022 Nov 21;2(4):e12118. doi: 10.1002/jcv2.12118 (PMC9937714; doi:10.1002/jcv2.12118)
Supplement: Supplementary file 1 — Supporting Information S1 [file JCV2-2-e12118-s001.docx]

# Supporting Information: Methods

## Participants by site

### Yale site.

Thirty-one female participants (at baseline age range 12-25.5, mean (standard deviation (SD))=18.2(3.2) years; time between visits range 1.2-5.5, mean(SD)=2.9(1.3) years) were recruited from the Yale School of Medicine medical center and greater Connecticut community. Participants met criteria for lifetime bipolar disorder (BD) or major depressive disorder (MDD; participants with BD n=32, 84%) according to the Diagnostic and Statistical Manual of Mental Disorders IV (Fourth ed. Text Revision; DSM-IV-TR) (Association, 2000); diagnoses were confirmed using the Structured Clinical Interview for DSM-IV (SCID) (First, Spitzer, Gibbon, & Williams, 1994) for participants ages ≥18 years and revised schedule for Affective Disorders and Schizophrenia for School-Age Children-Present and Lifetime Version (K-SADS-PL) (Kaufman et al., 1997) for participants <18 years. Participants with a history of alcohol or substance abuse or dependence were included due to high comorbidity with mood disorders (Kessler et al., 1997). All participants, except one, did not meet criteria for alcohol/substance abuse/dependence for >3 months (one participant had both alcohol dependence >2 weeks and cannabis dependence >1 month). Exclusion criteria were history of medical or neurological conditions that could affect the brain, except treated hypothyroidism for two participants, and magnetic resonance imaging (MRI) contraindications. Participants above 18 years provided written informed consent, while participants below 18 years provided written informed assent and their parent/guardian provided written informed permission. The study was approved by the Yale School of Medicine Human Investigation Committee/Institutional Review Board. Data of 1-25% of participants related to brain volume, although not cortical thickness or surface area measures were, included elsewhere (Chepenik et al., 2012; Edmiston et al., 2011; Fan et al., 2019; Johnston et al., 2017; Kalmar et al., 2009; Elizabeth Thomas Cox Lippard et al., 2017; Lippard et al., 2019; Elizabeth T. C. Lippard et al., 2017; Najt et al., 2016; Wang, Jackowski, et al., 2008; Wang, Kalmar, et al., 2008; Wang et al., 2009; Wang et al., 2011; Weathers et al., 2018; Womer et al., 2009).

### Cambridge site.

Sixty-one female participants (at baseline age range 12-17, mean(SD)=15.1(1.3) years; time between visits range 1-1.6, mean(SD)=1.6(0.2) years; all MDD) were a subset from an adjunct study from a larger clinical trial (reference 09/H0308/137) (Goodyer et al., 2017a, 2017b; Goodyer et al., 2011; Hagan et al., 2013). Participants met criteria for current moderate or severe MDD according to the DSM-IV criteria, which were confirmed by both child and parent interview using the K-SADS-PL (Kaufman et al., 1997). Participants completed the Mood and Feelings Questionnaire resulting in a score of 27 or more out of a possible range of 0-66 (Angold et al., 1987). Exclusion criteria were alcohol or substance dependence, generalized learning problems (assessed by clinicians) or a pervasive developmental disorder that results in an inability to complete the questionnaires (assessed by clinicians and confirmed by researchers), pregnancy or breastfeeding or currently having sexual relations without reliable contraception, brain abnormality determined by a consultant radiologist specializing in neuroanatomy, MRI contraindications, and current use of medication that may adversely interact with a selective serotonin reuptake inhibitor (Goodyer et al., 2017a; Hagan et al., 2013). Adolescents gave informed written assent and parent/carer gave informed written consent. The study was approved by the National Research Ethics Service Committee East of England- Cambridge Central. Data of 66% of participants were included in previous publications (Hagan et al., 2015; Villa et al., 2020).

## Gray matter structural imaging and diffusion tensor imaging data acquisition

Yale.

Imaging data were acquired with a 3 Tesla Siemens Trio scanner (Siemens, Erlangen, Germany). A high-resolution, three-dimensional Magnetization Prepared Rapid Acquisition Gradient Echo (3D-MPRAGE) T1-weighted sequence was used to acquire images in the sagittal plane with parameters: 160 slices of 1 mm without gap with two averages, repetition time (TR)=1500 ms, echo time (TE)=2.83 ms (n=16, 52%) or 2.77 ms (n=15, 48%), inversion time (TI)=700, flip angle=15°, matrix=256×256, field of view (FOV)=256×256 mm^2^, voxel size=1 mm^3^.

DTI data were acquired in alignment with the anterior commissure–posterior commissure plane with diffusion sensitizing gradients applied along 32 non-collinear directions with b value=1000 s/mm^2^ together with an acquisition without diffusion weighting (b value=0) and parameters: 40 slices of 3 mm without gap, TR=7400 ms, TE=115 ms, flip angle=90°, matrix=128×128, FOV=256×256 mm^2^, voxel size=3 mm^3^.

### Cambridge.

Imaging data were acquired with a 3 Tesla Magnetom Trio Tim scanner (Siemens, Erlangen, Germany). 3D-MPRAGE T1-weighted sequences was used to acquire images in the sagittal plane with parameters: 176 slices of 1 mm without gap, TR=2300 ms, TE=2.98 ms, TI=900 ms, flip angle=9˚, matrix=240×256, FOV=240×256 mm^2^, voxel size=1 mm^3^, series=interleaved.

DTI data were acquired with thirteen diffusion-weighted directed volumes and 5 volumes without diffusion-weighting (b=0) and parameters: 63 slices of 2 mm, TR=8300 ms, TE=98 ms, flip angle=90°, matrix=96×96, FOV=192×126 mm^2^, voxel size=2 mm^3^, series=interleaved.

# References

Angold, A., Weissman, M. M., John, K., Merikancas, K. R., Prusoff, B. A., Wickramaratne, P., et al. (1987). Parent and child reports of depressive symptoms in children at low and high risk of depression. *Journal of Child Psychology and Psychiatry, 28*(6), 901-915.

Association, A. P. (2000). Diagnostic and Statistical Manual of Mental Disorders (4th ed., text rev.).

Chepenik, L. G., Wang, F., Spencer, L., Spann, M., Kalmar, J. H., Womer, F., et al. (2012). Structure–function associations in hippocampus in bipolar disorder. *Biological psychology, 90*(1), 18-22.

Edmiston, E. E., Wang, F., Kalmar, J. H., Womer, F. Y., Chepenik, L. G., Pittman, B., et al. (2011). Lateral ventricle volume and psychotic features in adolescents and adults with bipolar disorder. *Psychiatry Research: Neuroimaging, 194*(3), 400-402.

Fan, S., Lippard, E. T. C., Sankar, A., Wallace, A., Johnston, J. A. Y., Wang, F., et al. (2019). Gray and white matter differences in adolescents and young adults with prior suicide attempts across bipolar and major depressive disorders. *Journal of affective disorders, 245*, 1089-1097.

First, M. B., Spitzer, R. L., Gibbon, M., & Williams, J. B. W. (1994). Structured clinical interview for Axis I DSM-IV disorders. *New York: Biometrics Research*.

Goodyer, I. M., Reynolds, S., Barrett, B., Byford, S., Dubicka, B., Hill, J., et al. (2017a). Cognitive-behavioural therapy and short-term psychoanalytic psychotherapy versus brief psychosocial intervention in adolescents with unipolar major depression (IMPACT): a multicentre, pragmatic, observer-blind, randomised controlled trial. *Health Technology Assessment (Winchester, England), 21*(12), 1.

Goodyer, I. M., Reynolds, S., Barrett, B., Byford, S., Dubicka, B., Hill, J., et al. (2017b). Cognitive behavioural therapy and short-term psychoanalytical psychotherapy versus a brief psychosocial intervention in adolescents with unipolar major depressive disorder (IMPACT): a multicentre, pragmatic, observer-blind, randomised controlled superiority trial. *The Lancet Psychiatry, 4*(2), 109-119.

Goodyer, I. M., Tsancheva, S., Byford, S., Dubicka, B., Hill, J., Kelvin, R., et al. (2011). Improving mood with psychoanalytic and cognitive therapies (IMPACT): a pragmatic effectiveness superiority trial to investigate whether specialised psychological treatment reduces the risk for relapse in adolescents with moderate to severe unipolar depression: study protocol for a randomised controlled trial. *Trials, 12*(1), 1-12.

Hagan, C. C., Graham, J. M. E., Tait, R., Widmer, B., van Nieuwenhuizen, A. O., Ooi, C., et al. (2015). Adolescents with current major depressive disorder show dissimilar patterns of age-related differences in ACC and thalamus. *NeuroImage: Clinical, 7*, 391-399.

Hagan, C. C., Graham, J. M. E., Widmer, B., Holt, R. J., Ooi, C., Van Nieuwenhuizen, A. O., et al. (2013). Magnetic resonance imaging of a randomized controlled trial investigating predictors of recovery following psychological treatment in adolescents with moderate to severe unipolar depression: study protocol for Magnetic Resonance-Improving Mood with Psychoanalytic and Cognitive Therapies (MR-IMPACT). *BMC psychiatry, 13*(1), 1-12.

Johnston, J. A. Y., Wang, F., Liu, J., Blond, B. N., Wallace, A., Liu, J., et al. (2017). Multimodal neuroimaging of frontolimbic structure and function associated with suicide attempts in adolescents and young adults with bipolar disorder. *American journal of psychiatry*.

Kalmar, J. H., Wang, F., Chepenik, L. G., Womer, F. Y., Jones, M. M., Pittman, B., et al. (2009). Relation between amygdala structure and function in adolescents with bipolar disorder. *Journal of the American Academy of Child & Adolescent Psychiatry, 48*(6), 636-642.

Kaufman, J., Birmaher, B., Brent, D., Rao, U. M. A., Flynn, C., Moreci, P., et al. (1997). Schedule for affective disorders and schizophrenia for school-age children-present and lifetime version (K-SADS-PL): initial reliability and validity data. *Journal of the American Academy of Child & Adolescent Psychiatry, 36*(7), 980-988.

Kessler, R. C., Crum, R. M., Warner, L. A., Nelson, C. B., Schulenberg, J., & Anthony, J. C. (1997). Lifetime co-occurrence of DSM-III-R alcohol abuse and dependence with other psychiatric disorders in the National Comorbidity Survey. *Archives of general psychiatry, 54*(4), 313-321.

Lippard, E. T. C., Jensen, K. P., Wang, F., Johnston, J. A. Y., Spencer, L., Pittman, B., et al. (2017). Effects of ANK3 variation on gray and white matter in bipolar disorder. *Molecular psychiatry, 22*(9), 1345-1351.

Lippard, E. T. C., Johnston, J. A. Y., Spencer, L., Quatrano, S., Fan, S., Sankar, A., et al. (2019). Preliminary examination of gray and white matter structure and longitudinal structural changes in frontal systems associated with future suicide attempts in adolescents and young adults with mood disorders. *Journal of affective disorders, 245*, 1139-1148.

Lippard, E. T. C., Mazure, C. M., Johnston, J. A. Y., Spencer, L., Weathers, J., Pittman, B., et al. (2017). Brain circuitry associated with the development of substance use in bipolar disorder and preliminary evidence for sexual dimorphism in adolescents. *Journal of neuroscience research, 95*(1-2), 777-791.

Najt, P., Wang, F., Spencer, L., Johnston, J. A. Y., Lippard, E. T. C., Pittman, B. P., et al. (2016). Anterior cortical development during adolescence in bipolar disorder. *Biological psychiatry, 79*(4), 303-310.

Villa, L. M., Goodyer, I. M., Tait, R., Kelvin, R., Reynolds, S., Wilkinson, P. O., et al. (2020). Cognitive behavioral therapy may have a rehabilitative, not normalizing, effect on functional connectivity in adolescent depression. *Journal of affective disorders, 268*, 1-11.

Wang, F., Jackowski, M., Kalmar, J. H., Chepenik, L. G., Tie, K., Qiu, M., et al. (2008). Abnormal anterior cingulum integrity in bipolar disorder determined through diffusion tensor imaging. *The British Journal of Psychiatry, 193*(2), 126-129.

Wang, F., Kalmar, J. H., Edmiston, E., Chepenik, L. G., Bhagwagar, Z., Spencer, L., et al. (2008). Abnormal corpus callosum integrity in bipolar disorder: a diffusion tensor imaging study. *Biological psychiatry, 64*(8), 730-733.

Wang, F., Kalmar, J. H., He, Y., Jackowski, M., Chepenik, L. G., Edmiston, E. E., et al. (2009). Functional and structural connectivity between the perigenual anterior cingulate and amygdala in bipolar disorder. *Biological psychiatry, 66*(5), 516-521.

Wang, F., Kalmar, J. H., Womer, F. Y., Edmiston, E. E., Chepenik, L. G., Chen, R., et al. (2011). Olfactocentric paralimbic cortex morphology in adolescents with bipolar disorder. *Brain, 134*(7), 2005-2012.

Weathers, J., Lippard, E. T. C., Spencer, L., Pittman, B., Wang, F., & Blumberg, H. P. (2018). Longitudinal diffusion tensor imaging study of adolescents and young adults with bipolar disorder. *Journal of the American Academy of Child & Adolescent Psychiatry, 57*(2), 111-117.

Womer, F. Y., Wang, F., Chepenik, L. G., Kalmar, J. H., Spencer, L., Edmiston, E., et al. (2009). Sexually dimorphic features of vermis morphology in bipolar disorder. *Bipolar disorders, 11*(7), 753-758.

**Table S1** Table lists number of participants for each modality analysis, also divided by site.

|  | Number of participants | Age range | Age (mean(SD)) | Between visits time range | Between visits time (mean(SD)) | Diagnosis (BD; n, %) | *f*SBs/ *f*SIs/ *f*nonSIBs (n, %) |
| --- | --- | --- | --- | --- | --- | --- | --- |
| Total sample | 92 | 12-25.5 | 16.1(2.6) | 1-5.5 | 2(1) | 26 (28%) | 40 (43%)/ 33 (36%)/ 19 (21%) |
| Yale | 31 | 12-25.5 | 18.2(3.2) | 1.2-5.5 | 2.9(1.3) | 26 (84%) | 11 (35%)/ 13 (42%)/ 7 (23%)/ |
| Cambridge | 61 | 12-17 | 15.1(1.3) | 1-1.6 | 1.6(0.2) | / | 29 (47%)/ 20 (33%)/ 12 (20%)/ |
| sMRI sample | 91 | 12-25.5 | 16.1(2.6) | 1-5.5 | 2(1) | 26 (28%) | 40 (44%)/ 32 (35%)/ 19 (21%) |
| Yale | 30 | 12-25.5 | 18.2(3.3) | 1.2-5.5 | 2.9(1.3) | 26 (87%) | 11 (37%)/ 12 (40%)/ 7 (23%) |
| Cambridge | 61 | 12-17 | 15.1(1.3) | 1-1.6 | 1.6(0.2) | / | 29 (47%)/ 20 (33%)/ 12 (20%) |
| DTI sample | 88 | 12-25.5 | 16.2(2.6) | 1-5.5 | 2.1(1) | 26 (29%) | 37 (42%)/ 33 (37%)/ 18 (21%) |
| Yale | 31 | 12-25.5 | 18.2(3.2) | 1.2-5.5 | 2.9(1.3) | 26 (84%) | 11 (35%)/ 13 (42%)/ 7 (23%) |
| Cambridge | 57 | 12-17 | 15.2(1.3) | 1-1.6 | 1.6(0.2) | / | 26 (46%)/ 20 (35%)/ 11 (19%) |

Abbreviations: BD=bipolar disorder; DTI=diffusion-weighted tensor imaging; *f*nonSIBs=group with no *future* suicidal ideation and behaviors; *f*SBs=group with *future* suicidal behavior; *f*SIs=group with *future* suicidal ideation; n=number of participants; SD=standard deviation; sMRI=structural magnetic resonance imaging.

**Table S2** Main effect of groups and *post-hoc* comparisons for cortical surface area.

| Region | Statistics^A^ | *Post-hoc* comparisons^B^ |
| --- | --- | --- |
| Left frontal pole | F(2,85)=0.4, p_uncorrected_=.7, p_FDR_>.9 |  |
| Right frontal pole | F(2,85)=0.2, p_uncorrected_=.8, p_FDR_>.9 |  |
| Left medial orbitofrontal | F(2,85)=0.2, p_uncorrected_=.8, p_FDR_>.9 |  |
| Right medial orbitofrontal | F(2,85)=0.005, p_uncorrected_>.9, p_FDR_>.9 |  |
| Left lateral orbitofrontal | F(2,85)=0.7, p_uncorrected_=.5, p_FDR_>.9 |  |
| Right lateral orbitofrontal | F(2,85)=0.04, p_uncorrected_>.9, p_FDR_>.9 |  |
| Left inferior frontal gyrus pars orbitalis | F(2,85)=0.03, p_uncorrected_>.9, p_FDR_>.9 |  |
| Right inferior frontal gyrus pars orbitalis | F(2,85)=1.4, p_uncorrected_=.2, p_FDR_>.9 |  |
| Left inferior frontal gyrus pars opercularis^1^ | F(2,83)=2.3, p_uncorrected_=.1, p_FDR_>.9 |  |
| Right inferior frontal gyrus pars opercularis | F(2,85)=0.6, p_uncorrected_=.5, p_FDR_>.9 |  |
| Left inferior frontal gyrus pars triangularis^2^ | F(2,84)=0.9, p_uncorrected_=.4, p_FDR_>.9 |  |
| Right inferior frontal gyrus pars triangularis^2^ | F(2,84)=0.3, p_uncorrected_=.7, p_FDR_>.9 |  |
| Left superior frontal | F(2,85)=0.2, p_uncorrected_=.8, p_FDR_>.9 |  |
| Right superior frontal | F(2,85)=1.2, p_uncorrected_=.3, p_FDR_>.9 |  |
| Left rostral middle frontal | F(2,85)=0.1, p_uncorrected_=.9, p_FDR_>.9 |  |
| Right rostral middle frontal | F(2,85)=1.5, p_uncorrected_=.2, p_FDR_>.9 |  |
| Left caudal middle frontal | F(2,85)=2.8, p_uncorrected_=.066, p_FDR_=.5, ω_p_^2^=0.038 | *f*SBs vs *f*nonSIBs q=.4, g=-0.35;  *f*SIs vs *f*nonSIBs q=.065, g=-0.63;  *f*SBs vs *f*SIs q=.4, g=0.31 ^§1^ |
| Right caudal middle frontal | **F(2,85)=3.6**, **p_uncorrected_=.032**, p_FDR_=.5, **ω_p_^2^=0.054** | *f*SBs vs *f*nonSIBs q>.9, g=0.03;  *f*SIs vs *f*nonSIBs q=.13, g=-0.48;  ***f*SBs vs *f*SIs** **q=.04**, **g=0.64** ^§2^ |
| Left rostral anterior cingulate | F(2,85)=0.15, p_uncorrected_=.8, p_FDR_>.9 |  |
| Right rostral anterior cingulate | F(2,85)=0.04, p_uncorrected_>.9, p_FDR_>.9 |  |
| Left caudal anterior cingulate^2^ | F(2,84)=0.5, p_uncorrected_=.6, p_FDR_>.9 |  |
| Right caudal anterior cingulate | F(2,85)=0.09, p_uncorrected_=.9, p_FDR_>.9 |  |
| Left posterior cingulate | F(2,85)=1.4, p_uncorrected_=.2, p_FDR_>.9 |  |
| Right posterior cingulate | F(2,85)=0.5, p_uncorrected_=.6, p_FDR_>.9 |  |
| Left superior temporal gyrus | F(2,85)=0.4, p_uncorrected_=.6, p_FDR_>.9 |  |
| Right superior temporal gyrus | F(2,85)=1.1, p_uncorrected_=.3, p_FDR_>.9 |  |
| Left middle temporal gyrus | **F(2,85)=3.1**, **p_uncorrected_=.05**, p_FDR_=.5, **ω_p_^2^=0.044** | *f*SBs vs *f*nonSIBs q=.06, g=-0.58;  *f*SIs vs *f*nonSIBs q=.14, g=-0.65;  *f*SBs vs *f*SIs q=.9, g=-0.09 ^§3^ |
| Right middle temporal gyrus | F(2,85)=0.2, p_uncorrected_=.8, p_FDR_>.9 |  |
| Left temporal pole^2^ | F(2,84)=1.3, p_uncorrected_=.3, p_FDR_>.9 |  |
| Right temporal pole | F(2,85)=2.8, p_uncorrected_=.06, p_FDR_=.5, ω_p_^2^=0.038 | *f*SBs vs *f*nonSIBs q=.093, g=0.63;  *f*SIs vs *f*nonSIBs q=.8, g=0.19;  *f*SBs vs *f*SIs q=.2, g=0.39 ^§4^ |
| Left insula | F(2,85)=1.0, p_uncorrected_=.4, p_FDR_>.9 |  |
| Right insula | F(2,85)=0.03, p_uncorrected_>.97, p_FDR_>.9 |  |

^A^ Groups were compared with one-way analysis of co-variance (cortical surface area as dependent variable, group as independent variable and age, site and intracranial volume as covariates), and effect size was calculated using partial omega squared (ω_p_^2^).

^B^ *Post-hoc* multiple pairwise-comparison between the means of groups (*f*SBs, *f*SIs, *f*nonSIBs) were performed using Tukey honest significant differences, and effect sizes were calculated using Hedge’s g.

^1^ 2 participants were labeled as outliers in the Rosner test.

^2^ 1 participant was labeled as outlier in the Rosner test.

^§1^ Left caudal middle frontal: *post-hoc* test revealed higher surface area in *f*SIs compared to *f*nonSIBs.

^§2^ Right caudal middle frontal: *post-hoc* test revealed higher surface area in *f*SIs compared to *f*SBs; *f*nonSIBs were non-significant intermediate.

^§3^ Left middle temporal gyrus: *post-hoc* test revealed higher surface area in *f*SBs compared to *f*nonSIBs. *f*SIs were non-significant intermediate.

^§4^ Right temporal pole: *post-hoc* test revealed lower surface area in *f*SBs compared to *f*nonSIBs.

Abbreviations: *f*nonSIBs=group with no *future* suicidal ideation and behaviors; *f*SBs=group with *future* suicidal behaviors; *f*SIs=group with *future* suicidal ideation; p_FDR_=multiple comparisons correction using false discovery rate.

**Table S3** Main effect of groups and *post-hoc* comparisons for cortical thickness.

| Region | Statistics^A^ | *Post-hoc* comparisons^B^ |
| --- | --- | --- |
| Left frontal pole | F(2,86)=1.2, p_uncorrected_=.3, p_FDR_=.8 |  |
| Right frontal pole^1^ | F(2,85)=1.1, p_uncorrected_=.3 p=.3, p_FDR_=.8 |  |
| Left medial orbitofrontal | F(2,86)=1.0, p_uncorrected_=.3 p=.3, p_FDR_=.8 |  |
| Right medial orbitofrontal | F(2,86)=0.2, p_uncorrected_=.8 p=.3, p_FDR_=.9 |  |
| Left lateral orbitofrontal | F(2,86)=2.1, p_uncorrected_=.13 p=.3, p_FDR_=.6 |  |
| Right lateral orbitofrontal | F(2,86)=2.7, p_uncorrected_=.071, p_FDR_=.6, ω_p_^2^=0.037 | *f*SBs vs *f*nonSIBs q=.096, g=0.57;  *f*SIs vs *f*nonSIBs q=.1, g=0.60;  *f*SBs vs *f*SIs q>.9, g=-0.01 ^§1^ |
| Left inferior frontal gyrus pars orbitalis | **F(2,86)=4.4**, **p_uncorrected_=.015**, p_FDR_=.2, **ω_p_^2^=0.069** | *f*SBs vs *f*nonSIBs q=.2, g=0.44;  ***f*SIs vs *f*nonSIBs** **q=.012**, **g=0.86**;  *f*SBs vs *f*SIs q=.2, g=-0.40 ^§2^ |
| Right inferior frontal gyrus pars orbitalis | **F(2,86)=5.3**, **p_uncorrected_=.007**, p_FDR_=.2, **ω_p_^2^=0.087** | ***f*SBs vs *f*nonSIBs q=.022**, **g=0.72**;  ***f*SIs vs *f*nonSIBs** **q=.008**, **g=0.96**;  *f*SBs vs *f*SIs q=.8, g=-0.14 ^§3^ |
| Left inferior frontal gyrus pars opercularis | F(2,86)=0.5, p_uncorrected_=.6, p_FDR_=.9 |  |
| Right inferior frontal gyrus pars opercularis | F(2,86)=.1, p_uncorrected_=.9, p_FDR_=.9 |  |
| Left inferior frontal gyrus pars triangularis | F(2,86)=1.0, p_uncorrected_=.3, p_FDR_=.8 |  |
| Right inferior frontal gyrus pars triangularis | F(2,86)=1.6, p_uncorrected_=.2, p_FDR_=.7 |  |
| Left superior frontal | F(2,86)=0.4, p_uncorrected_=.7, p_FDR_=.9 |  |
| Right superior frontal | F(2,86)=0.06, p_uncorrected_=.9, p_FDR_>.9 |  |
| Left rostral middle frontal | F(2,86)=2.3, p_uncorrected_=.11, p_FDR_=.6 |  |
| Right rostral middle frontal | F(2,86)=2.1, p_uncorrected_=.13, p_FDR_=.6 |  |
| Left caudal middle frontal | F(2,86)=0.1, p_uncorrected_=.9, p_FDR_=.9 |  |
| Right caudal middle frontal | F(2,86)=2.0, p_uncorrected_=.14, p_FDR_=.6 |  |
| Left rostral anterior cingulate | F(2,86)=1.2, p_uncorrected_=.3, p_FDR_=.8 |  |
| Right rostral anterior cingulate | F(2,86)=0.5, p_uncorrected_=.6, p_FDR_=.9 |  |
| Left caudal anterior cingulate | F(2,86)=1.0, p_uncorrected_=.4, p_FDR_=.8 |  |
| Right caudal anterior cingulate | F(2,86)=0.3, p_uncorrected_=.7, p_FDR_=.9 |  |
| Left posterior cingulate | F(2,86)=0.1, p_uncorrected_=.8, p_FDR_=.9 |  |
| Right posterior cingulate | F(2,86)=0.01, p_uncorrected_>.9, p_FDR_>.9 |  |
| Left superior temporal gyrus | F(2,86)=0.3, p_uncorrected_=.7, p_FDR_=.9 |  |
| Right superior temporal gyrus | F(2,86)=0.6, p_uncorrected_=.5, p_FDR_=.9 |  |
| Left middle temporal gyrus | F(2,86)=0.9, p_uncorrected_=.4, p_FDR_=.8 |  |
| Right middle temporal gyrus | **F(2,86)=4.1**, **p_uncorrected_=.019**, p_FDR_=.2, **ω_p_^2^=0.065** | *f*SBs vs *f*nonSIBs q=.064, g=0.68;  ***f*SIs vs *f*nonSIBs** **q=.018**, **g=0.79**;  *f*SBs vs *f*SIs q=.8, g=-0.16 ^§4^ |
| Left temporal pole | F(2,86)=0.9, p_uncorrected_=.4, p_FDR_=.8 |  |
| Right temporal pole | F(2,86)=0.5, p_uncorrected_=.6, p_FDR_=.9 |  |
| Left insula | F(2,86)=0.2, p_uncorrected_=.8, p_FDR_=.9 |  |
| Right insula | F(2,86)=0.1, p_uncorrected_=.9, p_FDR_=.9 |  |

^A^ Groups were compared with one-way analysis of co-variance (cortical thickness as dependent variable, group as independent variable and age and site as covariates), and effect size was calculated using partial omega squared (ω_p_^2^).

^B^ *Post-hoc* multiple pairwise-comparison between the means of groups (*f*SBs, *f*SIs, *f*nonSIBs) were performed using Tukey honest significant differences, and effect sizes were calculated using Hedge’s g.

^1^ 1 participant was labeled as outlier in the Rosner test.

^§1^ Right lateral orbitofrontal: *post-hoc* test revealed lower cortical thickness in *f*SBs compared to *f*nonSIBs. *f*SIs were non-significant intermediate.

^§2^ Left inferior frontal gyrus pars orbitalis: *post-hoc* test revealed lower cortical thickness in *f*SIs compared to *f*nonSIBs; *f*SBs were non-significant intermediate.

^§3^ Right inferior frontal gyrus pars orbitalis: *post-hoc* test revealed lower cortical thickness in *f*SBs and *f*SIs compared to *f*nonSIBs.

^§4^ Right middle temporal gyrus: *post-hoc* test revealed lower cortical thickness in *f*SBs and *f*SIs compared to *f*nonSIBs.

Abbreviations: *f*nonSIBs=group with no *future* suicidal ideation and behaviors; *f*SBs=group with *future* suicidal behaviors; *f*SIs=group with *future* suicidal ideation; p_FDR_=multiple comparisons correction using false discovery rate.

**Table S4** Main effect of groups and *post-hoc* comparisons for subcortical grey volumes.

| Region | Statistics^A^ | *Post-hoc* comparisons^B^ |
| --- | --- | --- |
| Left amygdala^1^ | F(2,83)= 0.4, p_uncorrected_=.7, p_FDR_=.8 |  |
| Right amygdala | F(2,85)=0.2, p_uncorrected_=.8, p_FDR_=.8 |  |
| Left hippocampus | F(2,85)= 0.6, p_uncorrected_=.5, p_FDR_=.8 |  |
| Right hippocampus | F(2,85)=0.4, p_uncorrected_=.6, p_FDR_=.8 |  |
| Left caudate^2^ | F(2,84)=0.2, p_uncorrected_=.8, p_FDR_=.8 |  |
| Right caudate^2^ | F(2,84)=0.3, p_uncorrected_=.7, p_FDR_=.8 |  |
| Left putamen^2^ | F(2,84)=2.1, p_uncorrected_=.12, p_FDR_=.6 |  |
| Right putamen | F(2,85)=1.1, p_uncorrected_=.3, p_FDR_=.8 |  |
| Left thalamus^2^ | F(2,84)=2.6, p_uncorrected_=.08, p_FDR_=.6, ω_p_^2^=0.034 | *f*SBs vs *f*nonSIBs q=.084, g=0.61;  *f*SIs vs *f*nonSIBs q=.2, g=0.46;  *f*SBs vs *f*SIs q=.9, g=0.13 ^§1^ |
| Right thalamus^2^ | F(2,84)=0.5, p_uncorrected_=.6, p_FDR_=.8 |  |

^A^ Groups were compared with one-way analysis of co-variance (subcortical grey volume as dependent variable, group as independent variable and age, site and intracranial volume as covariates), and effect size was calculated using partial omega squared (ω_p_^2^).

^B^ *Post-hoc* multiple pairwise-comparison between the means of groups (*f*SBs, *f*SIs, *f*nonSIBs) were performed using Tukey honest significant differences, and effect sizes were calculated using Hedge’s g.

^1^ 2 participants were labeled as outliers in the Rosner test.

^2^ 1 participant was labeled as outlier in the Rosner test.

^§1^ Left thalamus: p*ost-hoc* test revealed lower grey volume in *f*SBs compared to *f*nonSIBs.

Abbreviations: *f*nonSIBs=group with no *future* suicidal ideation and behaviors; *f*SBs=group with *future* suicidal behaviors; *f*SIs=group with *future* suicidal ideation; p_FDR_=multiple comparisons correction using false discovery rate.

**Table S5** Main effect of groups and *post-hoc* comparisons for fractional anisotropy.

| Region | Statistics^A^ | *Post-hoc* comparisons^B^ |
| --- | --- | --- |
| Left corona radiata | **F(2,83)=3.9**, **p_uncorrected_=.023**, p_FDR_=.11, **ω_p_^2^=0.063** | ***f*SBs vs *f*nonSIBs q=.041**, **g=0.82**;  ***f*SIs vs *f*nonSIBs** **q=.032**, **g=0.68**;  *f*SBs vs *f*SIs q>.9, g=-0.04 ^§1^ |
| Right corona radiata^1^ | F(2,80)=2.4, p_uncorrected_=.099, p_FDR_=.21, ω_p_^2^=0.032 | *f*SBs vs *f*nonSIBs q=.091, g=0.59;  *f*SIs vs *f*nonSIBs q=.4, g=0.42;  *f*SBs vs *f*SIs q=.6, g=0.23 ^§2^ |
| Left internal capsule^1^ | F(2,80)=1.0, p_uncorrected_=.3, p_FDR_=.45 |  |
| Right internal capsule^1^ | F(2,80)=2.4, p_uncorrected_=.097, p_FDR_=.21, ω_p_^2^=0.032 | *f*SBs vs *f*nonSIBs q=.094, g=0.60;  *f*SIs vs *f*nonSIBs q=.2, g=0.49;  *f*SBs vs *f*SIs q=.9, g=0.12 ^§3^ |
| Left external capsule^1^ | F(2,80)=0.3, p_uncorrected_=.7, p_FDR_=.70 |  |
| Right external capsule | F(2,83)=1.5, p_uncorrected_=.2, p_FDR_=.36 |  |
| Left inferior fronto-occipital fasciculus^2^ | F(2,82)=2.3, p_uncorrected_=.11, p_FDR_=.21 |  |
| Right inferior fronto-occipital fasciculus | F(2,83)=1.1, p_uncorrected_=.3, p_FDR_=.45 |  |
| Left superior fronto-occipital fasciculus | F(2,83)=0.4, p_uncorrected_=.6, p_FDR_=.69 |  |
| Right superior fronto-occipital fasciculus^1^ | **F(2,80)=4.1**, **p_uncorrected_=.021**, p_FDR_=.11, **ω_p_^2^=0.067** | ***f*SBs vs *f*nonSIBs q=.019**, **g=0.83**;  *f*SIs vs *f*nonSIBs q=.12, g=0.57;  *f*SBs vs *f*SIs q=.7, g=0.20 ^§4^ |
| Left uncinate fasciculus | **F(2,83)=4.1**, **p_uncorrected_=.019**, p_FDR_=.11, **ω_p_^2^=0.067** | ***f*SBs vs *f*nonSIBs q=.024**, **g=0.77**;  ***f*SIs vs *f*nonSIBs** **q=.045, g=0.76**;  *f*SBs vs *f*SIs q>.9, g=0.05 ^§5^ |
| Right uncinate fasciculus | **F(2,83)=3.5, p_uncorrected_=.034**, p_FDR_=.12, **ω_p_^2^=0.054** | ***f*SBs vs *f*nonSIBs q=.032**, **g=0.77**;  *f*SIs vs *f*nonSIBs q=.12, g=0.57;  *f*SBs vs *f*SIs q=.8, g=0.15 ^§6^ |
| Cingulum | F(2,83)=0.6, p_uncorrected_=.5, p_FDR_=.64 |  |
| Corpus callosum^1^ | F(2,80)=2.1, p_uncorrected_=.13, p_FDR_=.23 |  |

^A^ Groups were compared with one-way analysis of co-variance (fractional anisotropy as dependent variable, group as independent variable and age and site as covariates), and effect size was calculated using partial omega squared (ω_p_^2^).

^B^ *Post-hoc* multiple pairwise-comparison between the means of groups (*f*SBs, *f*SIs, *f*nonSIBs) were performed using Tukey honest significant differences, and effect sizes were calculated using Hedge’s g.

^1^ 3 participants were labeled as outliers in the Rosner test.

^2^ 1 participant was labeled as outlier in the Rosner test.

^§1^ Left corona radiata: *post-hoc* test revealed lower fractional anisotropy in *f*SBs and *f*SIs compared to *f*nonSIBs.

^§2^ Right corona radiata: *post-hoc* test revealed lower fractional anisotropy in *f*SBs compared to *f*nonSIBs.

^§3^ Right internal capsule: *post-hoc* test revealed lower fractional anisotropy in *f*SBs compared to *f*nonSIBs; *f*SIs were non-significant intermediate.

^§4^ Right superior fronto-occipital fasciculus: *post-hoc* test revealed lower fractional anisotropy in *f*SBs compared to *f*nonSIBs; *f*SIs were non-significant intermediate.

^§5^ Left uncinate fasciculus: *post-hoc* test revealed lower fractional anisotropy in *f*SBs and *f*SIs compared to *f*nonSIBs.

^§6^ Right uncinate fasciculus: *post-hoc* test revealed lower fractional anisotropy in *f*SBs compared to *f*nonSIBs; *f*SIs were non-significant intermediate.

Abbreviations: *f*nonSIBs=group with no *future* suicidal ideation and behaviors; *f*SBs=group with *future* suicidal behaviors; *f*SIs=group with *future* suicidal ideation; p_FDR_=multiple comparisons correction using false discovery rate.

**Table S6** Main effect of groups after covarying for past lifetime SBs.

| Region | Statistics^A^ |
| --- | --- |
| Right caudal middle frontal surface area | F(2,83)=3.8, p_uncorrected_=.027, ω_p_^2^=0.058 |
| Left middle temporal gyrus surface area | F(2,83)=3.5, p_uncorrected_=.036, ω_p_^2^=0.052 |
| Left inferior frontal gyrus pars orbitalis cortical thickness | F(2,84)=4.6, p_uncorrected_=.012, ω_p_^2^=0.075 |
| Right inferior frontal gyrus pars orbitalis cortical thickness | F(2,86)=5.6, p_uncorrected_=.005, ω_p_^2^=0.094 |
| Right middle temporal gyrus cortical thickness | F(2,84)=5.1, p_uncorrected_=.008, ω_p_^2^=0.084 |
| Left corona radiata fractional anisotropy | F(2,81)=2.8, p_uncorrected_=.065, ω_p_^2^=0.04 |
| Right superior fronto-occipital fasciculus fractional anisotropy^1^ | F(2,78)=2.4, p_uncorrected_=.096, ω_p_^2^=0.033 |
| Left uncinate fasciculus fractional anisotropy | F(2,81)=3.1, p_uncorrected_=.052, ω_p_^2^=0.045 |
| Right uncinate fasciculus fractional anisotropy | F(2,81)=3.1, p_uncorrected_=.052, ω_p_^2^=0.045 |

^A^ Groups were compared with one-way analysis of co-variance (surface area, cortical thickness or fractional anisotropy as dependent variable, group as independent variable and age, site, ICV for surface area, only, and lifetime suicide behaviors as covariates) and effect size was calculated using partial omega squared (ω_p_^2^).

^1^ 3 participants were labeled as outliers in the Rosner test.

Abbreviations: *f*nonSIBs=group with no *future* suicidal ideation and behaviors; *f*SBs=group with *future* suicidal behaviors; *f*SIs=group with *future* suicidal ideation.
